# Legislation in Iowa

**Published:** 1896-02

**Authors:** 


### LEGISLATION IN IOWA.

THE Act which we print in this number of the JOURNAL, looking to increased efficiency and power in dealing with the questions involving a wholesome and pure milk- and meat-supply for Iowa, contains a number of features which are well worth considering by all those who contemplate inaugurating plans for better veterinary sanitary police systems in their States. We trust that our friends in Iowa may meet with success in their efforts, and we know if failure comes this time it will not be for lack of enthusiasm and work on the part of Iowa veterinarians, and we feel sure that their efforts will be finally crowned with success. A strong arm of support for the Iowa veterinarians has been found already in the influence and assistance of their medical brethren.

